# Usefulness of Dynamic Assessment of Clinical and Laboratory Factors in Severe Acute Pancreatitis

**DOI:** 10.3390/jcm13154412

**Published:** 2024-07-28

**Authors:** Marta Librero-Jiménez, Francisco Valverde-López, Patricia Abellán-Alfocea, María Carmen Fernández-Cano, Eleazar Fernández-Fernández, Juan Gabriel Martínez-Cara, Elisabet López-González, Rita Jiménez-Rosales, Eduardo Redondo-Cerezo

**Affiliations:** Department of Gastroenterology and Hepatology, University Hospital Virgen de las Nieves, 18014 Granada, Spain; marta.librero.sspa@juntadeandalucia.es (M.L.-J.); patricia.abellan.sspa@juntadeandalucia.es (P.A.-A.); mariac.fernandez.cano.sspa@juntadeandalucia.es (M.C.F.-C.); eleazar.fernandez.sspa@juntadeandalucia.es (E.F.-F.); juang.martinez.sspa@juntadeandalucia.es (J.G.M.-C.); elisabet.lopez.gonzalez.sspa@juntadeandalucia.es (E.L.-G.); ritaajimenezr@ugr.es (R.J.-R.); eredondoc@ugr.es (E.R.-C.)

**Keywords:** acute pancreatitis, predicting severity, BISAP, severe acute pancreatitis

## Abstract

**Background/Objectives:** Early identification of patients at risk of developing severe acute pancreatitis (SAP) is still an issue. Dynamic assessment of clinical and laboratory parameters within the first 48 h of admission may offer valuable insights into the prediction of unfavorable outcomes such as SAP and death. **Methods:** A prospective observational study was conducted on a cohort of patients admitted for AP at a tertiary referral hospital. Clinical and laboratory data were collected on admission and at 48 h. Patients were classified based on the Revised Atlanta classification. Logistic regression analysis was performed to identify independent risk factors for SAP. Likelihood ratios and post-test probabilities were calculated to assess the clinical usefulness of predictive markers. **Results:** 227 patients were included, with biliary etiology being the most common and a prevalence of SAP and death of 10.7% and 5.7%, respectively. BISAP ≥ 2 on admission, presence of SIRS after 48 h, rise in heart rate over 20 bpm, and any increase in BUN after 48 h were independent risk factors for SAP. The combination of these factors increased the post-test probability of SAP and death, with BISAP ≥ 2 combined with the presence of SIRS after 48 h showing the highest probability (82% and 73%, respectively). **Conclusions:** Dynamic assessment of BUN, heart rate, and SIRS within the first 48 h of admission can aid in predicting the development of SAP and death in patients with AP. These findings underscore the importance of continuous monitoring, although multicenter studies are warranted to refine predictive models for SAP.

## 1. Introduction

Acute pancreatitis (AP) is one of the most common disorders leading to admission in gastroenterology departments, with incidence rates over 34 patients per 100,000 individuals in North America and Western Pacific Regions and as high as 72 patients per 100,000 in Spain [1,2]. Overall mortality rate has been estimated at 4.2%, but in cases of severe acute pancreatitis (SAP), mortality increases dramatically up to 50% [3]. However, following the revised Atlanta classification, most patients cannot be classified into mild, moderate, or severe among the first 48 h or even up to 96 h [4,5]. Although several scoring systems, such as Acute Physiology and Chronic Health Evaluation (APACHE II), Bedside Index of Severity in Acute Pancreatitis (BISAP) or Systemic Inflammatory Response Syndrome (SIRS), have been used to predict SAP in the early moments of the disease [6,7,8], all of them have shown little value in clinical practice due to their low post-test probability [9]. Furthermore, although there is currently no specific treatment for AP, we need tools to accurately identify and select patients at risk of unfavorable outcomes for inclusion in interventional clinical trials [4].

Although early prediction upon admission would be ideal, currently it seems unlikely to develop a marker that accurately predicts severity so early. This is probably due to the dynamic molecular, immune, and local events that lead to systemic inflammation and organ failure throughout the course of the disease. These events are often absent at their onset and subject to individual differences attributable to genetic predisposition [10,11]. Hence, a dynamic assessment based on the identification of early changes in clinical and laboratory parameters within the first 48 h could prove valuable in detecting unfavorable outcomes or be used in future scores.

The aim of our study was to assess changes in clinical and laboratory parameters used in daily clinical practice in AP, as well as markers routinely used in the first 48 h of admission, to seek out independent risk factors for SAP. Additionally, we sought to analyze how these parameters impact the prediction of severity and mortality in real practice.

## 2. Materials and Methods

### 2.1. Study Design

A prospective observational study on a cohort of consecutive patients admitted for AP treatment in our tertiary referral hospital was designed. All AP patients were included between March and November 2017, and clinical diagnosis was made following Revised Atlanta Classification [5]. Patients younger than 18 years old and those who refused to sign informed consent were excluded. The STROBE statements were used to ensure proper reporting of methods, results, and discussion.

### 2.2. Data Collection

Diagnosis of AP was made in patients addressing 2 of these 3 criteria: abdominal pain consistent with the disease, increased serum amylase or lipase levels at least 3 times the upper normal limit, and characteristic findings in abdominal imaging such as ultrasonography or computed tomographic (CT) scan [12]. Blood tests including hematological and biochemical parameters, such as blood urea nitrogen (BUN), creatinine (Cr), or C-reactive protein (CRP), among others routinely used in AP, were performed on admission and after 48 h, while procalcitonin and lactate (measured by gasometry) were performed only on admission. Clinical features such as blood pressure, heart rate, and pulse oximetry were collected on admission and after 48 h. Dynamic changes in clinical and laboratory parameters known to be related to SAP, such as BUN or hematocrit during the first 48 h, but also others poorly studied, such as CRP, total bilirubin (TB), and heart rate, were also collected [13]. Any rise in laboratory values within the initial 48 h was considered (increasing BUN, hematocrit, CRP, TB), while heart rate increases of over 10 and 20 beats per minute (bpm) were taken into account due to daily variability. Systemic Inflammatory Response Syndrome (SIRS) was calculated on admission and after 48 h. Patients addressing two or more of the following were considered to present SIRS: body temperature over 38 or under 36 degrees Celsius, heart rate > 90 bpm, respiratory rate > 20 breaths per minute or partial pressure of CO_2_ < 32 mmHg, leukocyte count > 12,000 × 10^9^ or < 4000 × 10^9^/L. BISAP was calculated on admission, adding one point when fulfilling any of the following items: BUN > 25 mg/dl, impaired mental status, presence of SIRS, age > 60 years, pleural effusion [6,14].

Epidemiological and clinical information (sex, age, current or past smoking habit or previous diseases) and ICU admission were also collected. Every patient underwent abdominal ultrasonography for etiological diagnosis. Patients without improvement during the first 5–7 days despite conservative management underwent a CT scan [5,12,15]. Regarding management, every patient started with moderate fluid resuscitation therapy based on Ringer’s lactate or sodium chloride solutions by the time of admission (approximately 2500–3000 mL/24 h), with adjustments made based on comorbidities, urinary outputs, and other clinical or laboratory signs of hypovolemia or fluid overload, as defined in our clinical protocol following main guidelines [12,15]. Patients were followed until discharge or death, and they were classified in mild, moderately severe, or SAP according to the Revised Atlanta classification [5]. Since SAP includes the majority of mortality, we divided patients in two groups for comparisons: one including patients with SAP and the other with the remaining (mild to moderately severe AP).

### 2.3. Data Analysis

Statistical analysis was carried out using PAWS Statistics 17.0 software (SPSS Inc., Chicago, IL, USA). Baseline characteristics, including laboratory markers, clinical features, comorbidities, score points, need for a CT scan, hospital stay, and outcomes of interest (death, ICU admission, necrotizing pancreatitis), were compared between the two groups using the chi-square test, Fisher’s exact test, or the Student’s *t*-test as appropriate. Nonparametric tests, such as U de Mann-Whitney, were used when necessary, and differences were considered statistically significant if *p* < 0.05. Receiver-operating characteristic curves (ROC) and area under the curve (AUC), along with standard error and 95% confidence intervals, were calculated for markers related to SAP in the bivariate analysis to select the best cutoff value for multivariate analysis. Thus, multivariate analysis based on multiple backward stepwise was used to seek out independent risk factors of SAP. Variables with *p* values > 0.05 were excluded, whereas variables with *p* values < 0.05 were included in the multivariate analysis design. Sensitivity, specificity, positive and negative likelihood ratio (+LR and −LR respectively), and the post-test probability of the factors independently related to SAP and other parameters who have been previously related in the literature were estimated using an open-access online calculator (http://araw.mede.uic.edu/cgi-bin/testcalc.pl (accessed on 19 June 2024)). Pretest probability was established based on the prevalence of SAP in our sample.

### 2.4. Ethics

Our study protocol was approved by the local ethics committee (protocol code: DIG-HVN 26012011, approved on 26 January 2011), and informed consent was obtained from each enrolled patient.

## 3. Results

We collected demographic, laboratory, and clinical data from 227 patients with a diagnosis of AP in a tertiary hospital. No patient refused to sign consent, so all patients were included. Baseline characteristics and comparisons between patients with SAP and mild to moderately severe AP are shown in Table 1. The mean age was 65.3 years old, and 112 patients were male (49.3%), and biliary etiology was the most common (67.8%). We did not find differences in sex distribution between both severity groups, but patients with SAP were older than those with mild to moderately severe AP.

Regarding scores on admission, BISAP ≥ 2 was related to SAP (*p* < 0.001) as well as the presence of SIRS on admission and SIRS after 48 h (both with *p* < 0.001). When assessing changes in clinical features and blood parameters after 48 h, any increase in BUN levels and a heart rate over 20 beats per minute (bpm) were significantly related to SAP (*p* < 0.001 and *p* = 0.02, respectively).

Patients who developed SAP had a higher likelihood of dying, ICU admission, CT scan performance, and necrotizing pancreatitis (all of them with *p* < 0.001).

### 3.1. Laboratory Parameters and Scores in Predicting SAP

AUC and the best cutoff values for laboratory markers and BISAP on admission were included for SAP prediction, as shown in Table 2. Only the AUC of leucocytes on admission did not reach statistic signification for SAP prediction (AUC 0.62; CI 95% 0.49–0.75; *p* = 0.06).

### 3.2. Logistic Regression

Once the best cutoff values were obtained based on ROC curves, logistic regression was performed with every marker significantly related to SAP in the bivariate analysis. Initially, BISAP ≥ 2, SIRS on admission, SIRS after 48 h, lactate ≥ 2.05 mEq/L, procalcitonin ≥ 0.32 ng/dL, glucose ≥ 130 mg/dL, creatinine ≥ 1.3 mg/dL, BUN ≥ 28 mg/dL, neutrophils ≥ 84.7%, neutrophils after 48 h ≥ 83.4%, leucocytes after 48 h ≥ 12.735 × 10^9^/L, BUN after 48 h ≥ 30 mg/dL, CRP after 48 h ≥ 280 mg/dL, rising BUN, heart rate increased over 20 bpm with respect to previous value, were included in the logistic regression. Eventually, risk factors with statistical significance were BISAP ≥ 2 on admission, SIRS after 48 h, rise in heart rate over 20 bpm, and any rise in BUN after 48 h (Table 3).

### 3.3. Clinical Usefulness of Severity Risk Factors Based on Post-Test Probability of SAP and Death

**Figure 1:**
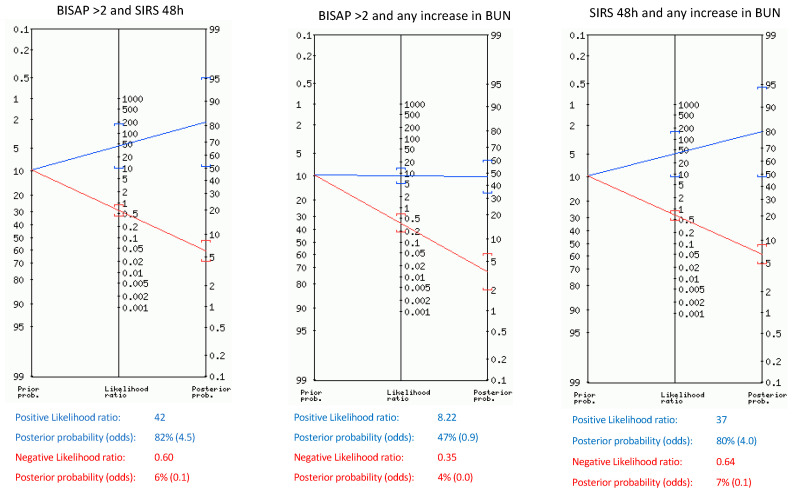
Positive, negative likelihood ratio, and post-test probability of SAP of combinations of parameters found to be independently related to SAP.

Likelihood ratios for SAP and death for single parameters and their combinations within the first 48 h are shown in Table 4, Table 5, and Table 6. The pretest probability of SAP and death in our sample was 10.7% and 5.7%, respectively. Upon admission, BISAP ≥ 2 and SIRS showed only a post-test probability of 23%, but after 48 h, SIRS and BUN ≥ 30 mg/dL showed the highest post-test probability of SAP (56% and 46%, respectively). Any rise in BUN and a rise in heart rate over 20 bpm during the first 48 h slightly increased the probability of SAP from 10.7% to 28% and 26%, respectively, whereas any rise in hematocrit only reached a post-test probability of SAP of 15%. When combining the parameters independently related to SAP (Figure 1), we found that BISAP ≥ 2 combined with persistent SIRS after 48 h and any rise in BUN combined with persistent SIRS showed the highest post-test probabilities (82% and 80%, respectively). Furthermore, those combinations rose the probability of death from 5.7% to 73% and 70%, respectively.

## 4. Discussion

Our study analyzed a representative sample of patients with AP in Spain in order to identify dynamic clinical and laboratory markers on admission and among the first 48 h for developing SAP. We conclude that any rise in BUN, an increase in heart rate over 20 bpm throughout the first 48 h, BISAP ≥ 2 on admission, and the presence of SIRS after 48 h were all independent risk factors for SAP. Furthermore, we assess how they change the probability of SAP and death based on our prevalence.

The dynamic assessment and monitoring of clinical and laboratory signs during the early course of the disease have been proposed as valuable predictors of severity [16,17,18]. Koutroumpakis et al. found that hematocrit > 44% and an increase in BUN during the first 24 h of admission outperformed APACHE II in predicting persistent organ failure [17], whereas Pando et al. only found changes in BUN during the first 24 h, but not hematocrit, to be reliable predictors such as BISAP and APACHE II in predicting mortality [16]. In our sample, any rise in BUN and an increase of heart rate over 20 bpm, but not changes in hematocrit, were significantly related to SAP on univariate and multivariate analyses. Furthermore, we also provide data about their real clinical performance, finding that any rise in BUN and an increase in HR > 20 bpm only improve the probability of SAP from 10.7% to 26–28% but up to 50% when both were present. Thus, although they are of little use as single markers, when combined, they provided valuable information about patient prognosis.

The role of the main scores on admission in predicting SAP has recently been assessed by Capurso et al. in a meta-analysis in which they found that no one reached a post-test probability higher than 50%, suggesting that novel approaches such as monitoring dynamic changes during the course of the disease could be helpful [9]. In fact, the most recent American College of Gastroenterology guidelines about AP management suggest classifying patients into low and high risk of developing SAP based on several factors such as BUN, SIRS, comorbidities, or obesity rather than relying solely on scores or single markers [4]. In the aforementioned meta-analysis, the prevalence of SAP ranges from 16–25%, which is higher than ours (10.7%), but it includes studies from different countries. Our prevalence and etiological distribution are similar to others previous Spanish studies, which found a prevalence of SAP of less than 7%, with biliary etiology being the main one, accounting for approximately 60% of cases [3,19]. In our sample, parameters on admission such as BISAP ≥ 2 and SIRS only showed a post-test probability of 23%, consistent with the findings of Capurso et al., given our even lower pre-test probability [9]. However, when present, SIRS after 48 h shows the highest post-test probability of SAP, reaching 56%. This reinforces the close relationship between the persistence or new onset of SIRS during the early moments of the disease and organ failure [20]. Furthermore, in patients who exhibit SIRS after 48 h and any increase in BUN (even when normal values), the post-test probability of SAP and death increases up to 80% and 70%, respectively. These data strongly suggest that close monitoring of SIRS and BUN can provide objective information regarding worsening within the first 48 h of the disease. The combination of known scores with laboratory or clinical markers for predicting SAP has been scarcely assessed. A recent Chinese retrospective study, with a prevalence of SAP of 26%, combined BISAP with laboratory parameters such as CRP and neutrophil-lymphocyte ratio, showing a high AUC when predicting SAP, but it does not mention how it influences the individual probability of SAP [21]. In our study, we evaluated the combination of BISAP with dynamic assessment of BUN, SIRS, and heart rate. We found that when BISAP ≥ 2, along with any increase in BUN or the presence of SIRS after 48 h, the individual risk of SAP increased considerably to 43% and 82%, respectively, while the probability of death rose to 25% and 73%, respectively.

Our study has several limitations. Firstly, being a single-center study and considering the variability in SAP index among different studies, our results may not be broadly generalizable. However, the findings regarding dynamic changes in laboratory and clinical parameters are consistent with previous reports [16,17]. Secondly, the majority of patients in our sample had a biliary etiology. While this is consistent with other Spanish cohorts, the results may not be broadly applicable to other countries where alcoholic or hyperlipidemic etiologies are predominant.

## 5. Conclusions

In conclusion, changes in BUN, heart rate, and particularly the presence of SIRS after 48 h are features that raise the probability of SAP and death. Additionally, patients with a BISAP score ≥ 2 upon admission who develop any of these factors during the first 48 h are at a high risk of developing SAP and death in our cohort. Multicenter studies assessing the global prevalence of SAP and death in each region and analyzing dynamic factors during the early phase of the disease can be helpful in determining which factors are most useful in assessing severity.

## Figures and Tables

**Table 1:** Patients’ characteristics and comparisons between patients who developed severe acute pancreatitis and mild to moderate acute pancreatitis.

	Total	Mild–Moderately Severe	Severe	*p*
N (%)	227	205 (90.4)	22 (10.7)	
Age, mean (SD)	65.3 (18.9)	64.5 (19.2)	74.5 (11.9)	0.01
Male, *n* (%)	112 (49.3)	99 (48.3)	13 (59.1)	n.s
Etiology				
- Biliary, *n* (%)	154 (67.8)	137 (66.8)	17 (77.3)	n.s
- Alcoholic, *n* (%)	22 (9.6)	22 (10.7)	0 (0)	n.s
- Idiopathic, *n* (%)	24 (10.5)	20 (9.8)	4 (18.1)	n.s
- Hypertriglyceridemia, *n* (%)	4 (1.7)	4 (1.9)	0 (0)	n.s
Systolic blood pressure, mean (SD), mmHg	134 (25)	139 (69)	133 (31)	n.s
Diastolic blood pressure, mean (SD), mmHg	73 (13)	73 (13)	74 (16)	n.s
Heart rate (SD), bpm	78 (17)	78 (16)	83 (25)	n.s
Oxygen pulse oxymetry (SD), %	95 (2)	95 (2)	94 (3)	n.s
Scores				
- BISAP ≥ 2, *n* (%)	82 (36.1)	63 (30.7)	19 (86.3)	<0.001
- BISAP ≥ 3, *n* (%)	26 (11.4)	19 (9.2)	7 (31.8)	0.02
- SIRS, *n* (%)	52 (22.9)	40 (19.5)	12 (54.5)	<0.001
- SIRS 48 h, *n* (%)	18 (7.9)	8 (3.9)	10 (45.4)	<0.001
Comorbidities				
- Diabetes	44 (19.3)	38 (18.5)	6 (27.7)	n.s
- COPD	27 (11.9)	24 (11.7)	3 (13.6)	n.s
- Heart Failure	16 (7)	13 (6.3)	3 (13.6)	n.s
- Arterial Hypertension	124 (54.6)	104 (50.7)	20 (91)	<0.001
- Hyperuricemia	20 (8.8)	15 (7.3)	5 (22.7)	0.03
- Dyslipidemia	59 (26)	52 (25.3)	7 (31.8)	n.s
- Renal Failure	19 (8.3)	15 (7.3)	4 (18.2)	0.09
Laboratory findings, mean (SD)				
Hematocrit (%)	43.2 (25.1)	43.54 (26.3)	40.22 (10.7)	n.s
Hematocrit 48 h (%)	38.4 (5.6)	38.34 (5.12)	38.78 (8.57)	n.s
Leukocytes, × 10^9^/L	13,191 (5382)	12,805 (5081)	16,308 (7497)	0.04
Leukocytes 48 h, × 10^9^/L	10,808 (5917)	10,111 (4716)	16,993 (10,493)	0.006
Neutrophils (%)	80 (10)	80 (10)	85 (6)	0.02
Neutrophils 48 h (%)	74 (12)	73 (12)	81 (10)	0.002
Glucose, mg/dL	137 (55)	132.43 (46.66)	179.27 (95.99)	0.03
ALT, IU/L	163 (213)	160 (217)	189 (116)	n.s
AST, IU/L	177 (200)	174 (204)	201 (118)	n.s
ALP (SD), IU/L	150 (133)	149 (135)	167 (107)	n.s
Bilirubin (SD), mg/dL	2 (2)	1.92 (1.9)	2.64 (2.5)	n.s
Amylase, IU/L	1358 (1138)	1310 (1089)	1829 (1472)	n.s
Triglycerides, mg/dL	173 (448)	177 (468)	129 (59)	n.s
Albumin, mg/dL	3.9 (0.6)	4.02 (0.76)	3.76 (0.51)	n.s
Procalcitonin (SD), ng/dL	3.3 (12.7)	2.26 (8.36)	12.11 (29.84)	0.01
CRP, mg/L	51.6 (80)	47 (73)	89 (128)	n.s
CRP 48 h, mg/L	150 (123)	137 (112)	272 (156)	0.001
Calcium, mg/dL	9.1 (0.7)	9.1 (0.6)	8.9 (0.8)	n.s
Calcium 48 h, mg/dL	8.6 (0.8)	8.6 (0.8)	8.4 (0.6)	n.s
Creatinine, mg/dL	1.17 (1.11)	1.10 (1.02)	2.22 (2.00)	0.01
Creatinine 48 h, mg/dL	1.10 (1.02)	0.98 (0.93)	2.18 (1.18)	<0.001
Lactate, mEq/L	1.95 (1.02)	1.88 (1.02)	2.44 (0.94)	0.02
Fibrinogen, mg/dL	429 (174)	431 (177)	408 (141)	n.s
BUN, mg/dL	22 (13)	20 (11)	38 (22)	0.01
BUN 48 h, mg/dL	20 (15)	16.95 (11.15)	44.26 (20.06)	<0.001
Dynamic parameters in 48 h, *n* (%)				
- Rising BUN	54 (29.7)	38 (23.6)	16 (76.2)	<0.001
- Rising Hematocrit	39 (18.2)	33 (17.1)	6 (27.2)	n.s
- Rising heart rate	74 (37.9)	65 (36.9)	9 (47.3)	n.s
- Rising HR > 20 bpm	21 (10.7)	15 (8.5)	6 (31.6)	0.02
- Rising CRP	174 (85.2)	157 (85.3)	17 (85)	n.s
- Rising TB	69 (30.3)	59 (28.8)	10 (45.4)	n.s
CT scan, *n*(%)	66 (29.1)	52 (25.3)	14 (63.6)	<0.001
Necrotizing pancreatitis, *n* (%)	18 (7.9)	11 (5.3)	7 (31.8)	<0.001
Death, *n* (%)	13 (5.7)	1 (0.5)	12 (54.5)	<0.001
**ICU admission, *n* (%)**	**15 (6.7)**	**7 (3.4)**	**8 (31.8)**	**<0.001**

SD: standard deviation; BISAP: Bedside Index for Severity in Acute Pancreatitis. COPD: chronic obstructive pulmonary disease. ALT: Alanine Aminotransferase. AST: aspartate aminotransferase. ALP BPM: beats per minute. ALP: Alkaline Phosphatase. SIRS: Systemic Inflammatory Response Syndrome. CRP: C-reactive protein. BUN: blood urea nitrogen. CT: computed tomography. TB: total bilirubin. ICU: intensive care unit. N.s: non statistically significant.

**Table 2:** AUC and best cut-off values of scoring systems and biomarkers in predicting severe acute pancreatitis.

Severe Acute Pancreatitis	AUC (95% CI)	Cut-Off Values
BISAP	0.8 (0.71–0.89)	≥2
Lactate	0.69 (0.56–0.81)	≥2.05 mEq/L
Procalcitonin	0.70 (0.59–0.80)	≥0.32 ng/dL
Glucose	0.66 (0.52–0.80)	≥130 mg/dL
Creatinine	0.83 (0.75–0.91)	≥1.3 mg/dL
BUN	0.82 (0.74–0.90)	≥28 mg/dL
Neutrophils	0.65 (0.55–0.76)	≥84.7%
Neutrophils 48 h	0.71 (0.58–0.83)	≥83.4%
Leucocytes 48 h	0.74 (0.62–0.86)	≥12.735 × 10^9^/L
Creatinine 48 h	0.89 (0.82–0.95)	≥1.57 mg/dL
BUN 48 h	0.91 (0.85–0.97)	≥30 mg/dL
CRP 48 h	0.75 (0.60–0.89)	≥280 mg/L

SAP: severe acute pancreatitis. AUC: Area under the curve. Blood urea nitrogen measured on admission. BUN 48 h: Blood urea nitrogen measured after 48 h. CRP 48 h: C-reactive protein measured after 48 h. Creatinine 48 h: Creatinine measured.

**Table 3:** Logistic regression model.

Predictor	OR	95% CI	*p*
Rising BUN	9.52	2.65–34.23	0.001
Rising HR > 20 bpm	5.80	1.29–25.98	0.022
SIRS 48 h	23.72	5.12–109.87	<0.001
BISAP ≥ 2	16.19	3.30–79.46	0.001

OR: Odds ratio. CI: confidence interval. BUN: blood urea nitrogen. HR: heart rate. BPM: beats per minute. SIRS: Systemic Inflammatory Response Syndrome. BISAP: Bedside Index for Severity in Acute Pancreatitis.

**Table 4:** Positive, negative likelihood ratio, and post-test probability of SAP of clinical and laboratory parameters related to SAP.

Predictor	Pre-Test Probability of SAP	+LR (CI 95%)	+Post-Test Probability of SAP	−LR (CI 95%)	−Post-Test Probability of SAP
Rise in heart rate > 20 bpm	10.7%	3.27 (1.44–7.43)	26% (13–44%)	0.79 (0.61–1.03)	8% (6–10%)
Rising BUN		3.57 (2.52–5.04)	28% (21–35%)	0.29 (1.13–0.63)	3% (1–6%)
Rising Hematocrit		1.59 (0.75–3.36)	15% (8–28%)	0.88 (0.67–1.14)	9% (7–12%)
BISAP ≥ 2		2.81 (2.16–3.66)	23% (19–28%)	0.20 (0.07–0.57)	2% (1–6%)
SIRS on admission		2.80 (1.74–4.48)	23% (16–32%)	0.56 (0.36–0.90)	6% (4–9%)
CRP ≥ 280 mg/dL after 48 h		4.43 (2.76–7.12)	33% (23–44%)	0.41 (0.23–0.75)	4% (2–8%)
BUN ≥ 30 mg/dL after 48 h		6.98 (4.29–11)	46% (34–57%)	0.27 (0.12–0.58)	3% (1–7%)
SIRS after 48 h		12 (5.11–26)	56% (36–74%)	0.57 (0.39–0.83)	6% (4–8%)

SAP: severe acute pancreatitis. LR: likelihood ratio. CI: confidence interval. BUN: blood urea nitrogen. BPM: beats per minute. SIRS: Systemic Inflammatory Response Syndrome. BISAP: Bedside Index for Severity in Acute Pancreatitis.

**Table 5:** Positive, negative likelihood ratio, and post-test probability of SAP of combinations of parameters found to be independently related to SAP.

Predictor	Pre-Test Probability of SAP	+LR (CI 95%)	+ Post-Test Probability of SAP	−LR (CI 95%)	− Post-Test Probability of SAP
BISAP ≥ 2 and rising BUN	10.7%	8.22 (4.80–14)	47% (34–60%)	0.35 (0.19–0.64)	4% (2–6%)
BISAP ≥ 2 and rising BUN and rise in heart rate > 20 bpm		14 (2.47–79)	60% (21–89%)	0.87 (0.74–1.03)	9% (7–10%)
BISAP ≥ 2 and SIRS after 48 h		42 (9.62–181)	82% (51–95%)	0.6 (0.42–0.85)	6% (4–8%)
Rise in heart rate > 20 bpm and rising BUN		9.32 (2.92–30)	50% (24–76%)	0.79 (0.63–0.99)	8% (6–10%)
SIRS after 48 h and rising BUN		37 (8.31–162)	80% (48–95%)	0.64 (0.47–0.88)	7% (5–9%)

SAP: severe acute pancreatitis. LR: likelihood ratio. CI: confidence interval. BUN: blood urea nitrogen. BPM: beats per minute. SIRS: Systemic Inflammatory Response Syndrome. BISAP: Bedside Index for Severity in Acute Pancreatitis.

**Table 6:** Negative and positive likelihood ratio, and post-test probability of death of clinical and laboratory parameters related to SAP.

Predictor	Pre-Test Probability of Death	+LR (CI 95%)	+Post-Test Probability of Death	−LR (CI 95%)	−Post-Test Probability of SAP
Rise in heart rate > 20 bpm	5.7%	2.47 (0.84–7.25)	13% (5–31%)	0.85 (0.63–1.15)	5% (4–7%)
Rising BUN		3.21 (2.19–4.71)	16% (12–22%)	0.30 (0.11–0.82)	2% (1–5%)
BISAP ≥ 2		2.27 (1.59–3.22)	12% (9–16%)	0.35 (0.13–0.95)	2% (1–5%)
SIRS after 48 h		21 (9.80–43)	56% (37–72%)	0.24 (0.09–0.65)	1% (1–4%)
BISAP ≥ 2 and rising BUN		5.51 (3.11–9.77)	25% (16–37%	0.43 (0.22–0.86)	3% (1–5%)
BISAP ≥ 2 and SIRS after 48 h		44 (13–146)	73% (44–90%)	0.39 (0.20–0.78)	2% (1–5%)
SIRS after 48 h and rising BUN		38 (11–130)	70% (40–89%)	0.47 (0.26–0.84)	3% (2–5%)

LR: likelihood ratio. CI: confidence interval. BUN: blood urea nitrogen. BPM: beats per minute. SIRS: Systemic Inflammatory Response Syndrome. BISAP: Bedside Index for Severity in Acute Pancreatitis.

## Data Availability

Data is unavailable due to privacy or ethical restrictions.
